# Effects of 2 Interventions on the Acceptability and Usability of a Sensing Glove for Measuring Force-Time Characteristics of Chiropractic Spinal Manipulative Therapy: A Crossover Study

**DOI:** 10.1016/j.jcm.2025.08.002

**Published:** 2025-09-10

**Authors:** Marie-Andrée Mercier, Martin Descarreaux, Andréanne K. Blanchette, Isabelle Pagé

**Affiliations:** a Department of Chiropratic, Université du Québec à Trois-Rivières, Trois-Rivières, Québec, Canada; b Department of Human Kinetics, Université du Québec à Trois-Rivières, Trois-Rivières, Québec, Canada; c School of Rehabilitation Sciences, Faculty of Medicine, Université Laval, Québec City, Québec, Canada; d Center for Interdisciplinary Research in Rehabilitation and Social Integration (Cirris), Centre Intégré Universitaire de Santé et de Services Sociaux de la Capitale-Nationale (CIUSSS-CN), Québec City, Québec, Canada

**Keywords:** Chiropractic, Conservative treatment, Complementary therapies, Biomechanical phenomena, Technology assessment, Biomedical

## Abstract

**Objectives:**

The objective of this study was to assess chiropractors’ and chiropractic students’ acceptability of a sensing glove system for measuring Spinal manipulative therapy (SMT) force-time characteristics and to compare the effectiveness of 2 interventions in improving their acceptability and perceived usability.

**Methods:**

Sixteen participants were recruited and initially rated their agreement with 12 statements about the acceptability of the sensing glove system. They then underwent 2 interventions designed to improve their acceptability of the system in a random order: a 7-minute informational video and a 20-minute supervised practice session. After each intervention, participants reassessed their agreement with the acceptability statements and provided feedback on the system’s usability through 10 additional statements. Data analysis used McNemar and Cochran’s Q tests to assess the interventions’ effectiveness.

**Results:**

The practice session led to a significantly higher number of participants agreeing with 4 more acceptability statements than at baseline, and with 1 more statement than after the video intervention. Conversely, the video intervention showed an increased proportion of agreement for only 1 statement compared to the baseline. Furthermore, the practice session resulted in a higher proportion of participants agreeing with 2 usability statements compared to the video intervention.

**Conclusions:**

The study highlights the importance of supervised hands-on practice in enhancing the acceptability and willingness of chiropractors and chiropractic students to adopt a force-sensing system in their clinical practice.

## Introduction

Spinal manipulative therapies (SMT), such as spinal manipulations and mobilizations, are treatment modalities commonly used by various healthcare professionals, including chiropractors, for the management of patients with musculoskeletal disorders.^1^, ^2^, ^3^ These therapies can be described in terms of their force-time characteristics (e.g., peak force, time-to-peak force), which appear to be, at least in part, related to the mechanisms underlying their clinical efficacy and safety.^4^^,^^5^ Over the years, several researchers have measured SMT force-time characteristics using devices located at the clinician-patient interface (e.g., using a load cell placed between the clinician’s hand and the patient’s back) or at the patient-table interface (e.g., using a force platform embedded in a treatment table).^6^ Given that current tools are more accessible, less cumbersome and easier to use,^6^ their usage rate is likely to increase in the coming years. Additionally, these devices now enable data collection in clinical practice contexts, which is crucial for enhancing external validity.^7^ Research across various biomechanical fields has shown that laboratory data often do not correspond to real-life applications (e.g.,^8^^,^^9^). Therefore, the use of sensing devices to measure the force-time characteristics of SMT administered by chiropractors in their practice could significantly deepen our understanding of SMT effects and safety, leading to improved patient care. For example, a scoping review on the effects of SMT frequency and dosage on neurophysiological and clinical outcomes recently highlighted a significant gap in the literature, with their inclusion of only 1 randomized controlled trial evaluating the effect of SMT force-time characteristics dosage on clinical outcomes.^5^ Similarly, how SMT dosage impacts the risk of musculoskeletal tissue injury remains largely unexplored.

A fundamental criterion for the successful implementation of the use of an SMT force-time characteristics measurement device in clinical practice would be the behavioral intention of chiropractors to use the technology with their patients. Theoretical models of implementation sciences have examined behavioral intention or willingness to use technology to explain how and why end users adopt a new technology.^10^^,^^11^ Acceptability and usability are among the most-frequently studied implementation outcomes, and are respectively defined as “the degree of primary users’ predisposition to carry out daily activities using the intended device” and “the extent to which a product can be used by specified users to achieve specified goals with effectiveness, efficiency, and satisfaction in a specified context of use.”^12^ Assessing a technology’s acceptability and usability early in the development process helps to identify barriers and document end-user needs, such as training and technical support requirements.^13^ In addition to providing the opportunity to adapt the technology specifications to better meet end users’ needs, implementation strategies such as acceptance-facilitating interventions can be developed.^14^

The framework on the determinants of perceived ease of use suggested by Venkatesh^15^ states that, in the absence of specific knowledge, an individual relies on general information to judge the technology, whereas when additional information is available, they will adjust their judgments without totally disregarding their initial judgment. Acceptability-facilitating interventions may therefore increase one’s intention to adopt and use a technology by modifying their perception of the technology’s ease of use and usefulness. Various acceptability-facilitating interventions have been shown to be effective, including providing individuals with information materials,^16^^,^^17^ the opportunity to experience the technology for a short period of time,^18^ or the demonstration of the technology or intervention through a short video.^19^^,^^20^

A recent scoping review identified factors that might limit or facilitate the use of a device measuring SMT force-time characteristics for research, educational and clinical purposes.^6^ Among the 46 included studies, none specifically assessed the acceptability or towards the use of such a device or its usability by clinicians. Nevertheless, the authors identified several factors that may influence the clinicians’ intention to use a device with their patients, such as potential loss of tactile sensation, device usability, feedback availability, versatility, clinician and patient comfort, cost and durability.^6^ How these factors specifically moderate the intention of clinicians, such as chiropractors, to use a device measuring their SMT force-time characteristics when treating patients remains unknown. Assessing acceptability and usability should provide relevant information to inform future developments and facilitate the development of strategies to promote the use of such devices by chiropractors.

Therefore, the objectives of this study were (1) to evaluate the acceptability of a sensing glove system measuring the SMT force-time characteristics by chiropractors and chiropractic students and (2) to compare the effectiveness of 2 acceptability-facilitating interventions on their acceptability and perceived usability towards this system. It was hypothesized that both interventions would improve acceptability and perceived usability, but that the practice session would have a greater impact due to the users’ experience with the system.

## Methods

### Design

This study is based on a mixed-method crossover design.

### Sensing Glove System

The sensing glove system evaluated in the present study was the Grip Versatek System from Tekscan (Boston, MA, USA). Each sensing glove is composed of 349 sensing elements divided into 18 regions: finger phalanges (distal, middle and proximal), thumb distal and proximal phalanges, metacarpophalangeal joints (divided into 2 regions), and thenar and hypothenar regions of the palm. Data were recorded using Tekscan’s pressure mapping software, which allows visualization and analysis of the pressure applied over the sensing glove, providing instantaneous feedback.

### Participants

To be included, participants were required to perform SMT to patients either as licensed chiropractors or as final-year chiropractic students (i.e., chiropractic interns) at the Université du Québec à Trois-Rivières (UQTR, Québec, Canada) outpatient chiropractic clinic. Participants were excluded if they presented any upper limb injury preventing them from performing manual therapies during the study, or if they had previous experience with a sensing glove system. All participants provided their written informed consent to take part in the study, in accordance with the UQTR Humans Research Ethics Board certification (CER-21-278-07.26). Using a computer-generated allocation sequence with a 1:1 ratio, participants were then randomly assigned to start with either the video or the practice session as their first intervention. A sample of 15 to 20 participants, with the aim of including an equal number of chiropractors and chiropractic students, was chosen due to the exploratory nature of this study, time constraints, and the number of potential participants available. Recruitment of participants and data collection took place between June and August 2021. This study was retrospectively registered on ClinicalTrials.gov (NCT06432946).

### Experiment

**Fig. 1:**
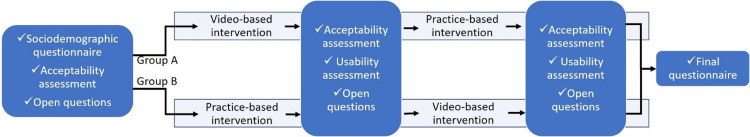
Study flow.

Participants were invited to take part in a 60-minute in-person session. They first completed an online questionnaire to provide their age, gender, and participant category. After reading an information sheet including the purpose of the study, a brief description of the 2 interventions and a photo of the sensing glove system, and using a personalized questionnaire, participants were asked to rate their baseline acceptability of using the system. Participants underwent the first of the 2 interventions, according to their assignment. Immediately following each intervention, the acceptability of using the system was evaluated de novo, as was their perception of its usability. After the second intervention, participants had to indicate which intervention should be prioritized in future research to facilitate the acceptability and perceived usability of the sensing glove system. They were also invited to describe how the interventions could be improved through open-ended questions. The experiment flow is shown in Figure 1.

### Acceptability-Facilitating Interventions

**Fig. 2:**
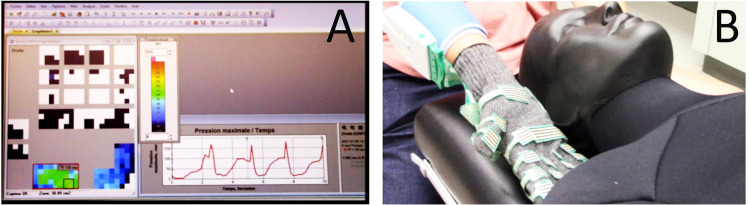
A. Real-time visual feedback provided to the participants during the practice session. B. Visualization of the sensing glove system and of the manikin used for the practice session.

The first facilitating intervention consisted of watching a 7-minute video demonstrating the sensing glove system, how it can be used to assess manual therapy biomechanics, and its relevance in research and clinical settings. The video was created by the research team using a PowerPoint presentation with voice-over. The PowerPoint presentation used for the video is available in the supplementary file. The other intervention consisted of a 20-minute practice session during which participants received verbal information about the sensing glove system, and were given the opportunity to perform palpation and manual therapy techniques (spinal mobilization/manipulation to the cervical and thoracic spine) on a human-sized manikin while wearing the sensing glove, and being guided by the researcher (Fig. 2). The information provided within the 7-minute video was also verbally provided during the practice session.

### Outcomes

#### General Opinion

At baseline, participants were asked to explain the reason(s) why they would be reluctant or unwilling to use the sensing glove system while treating patients. Following each intervention, they were asked whether their opinion had changed.

#### Acceptability

Participant’s acceptability of using the pressure-sensing glove system with their patients was assessed at baseline (prospective acceptability), and immediately after each intervention. For this purpose, a custom questionnaire was developed using 12 of the 27 statements of the extended version of the Canadian French Unified Theory of Acceptance and Use of Technology (UTAUT2) questionnaire.^21^ The validity and reliability of UTAUT and its extended version (UTAUT2) are well-supported by empirical research, demonstrating their effectiveness in explaining technology acceptance across different contexts.^22^ The UTAUT2 has been found to be a powerful predictor of behavioral intention and technology use.^23^ The UTAUT and UTAUT2 models can be used in whole or in part depending on the purpose and context of their use.^23^ Our research team cross-culturally adapted the original English version to Canadian French, although we did not performed a full psychometric evaluation of the translated version. Participants were asked to rate their level of agreement with each of the 12 statements using a 7-point Likert scale, the anchor points being “strongly agree” and “strongly disagree.” The statements were slightly modified at baseline to assess participants’ beliefs, considering that they had not yet undergone any of the facilitating interventions.

#### Usability

The pressure-sensing glove system perceived usability was assessed immediately after each intervention. It was not assessed at baseline, as it is a concept requiring a minimum of knowledge or experience with the technology. Participants were required to rate their level of agreement with the 10 statements of the Canadian French version of the System Usability Scale (F-SUS).^24^ The SUS and its Canadian French translation have proven to be reliable and valid tools for assessing the perceived usability of a system or technology.^24^^,^^25^ The F-SUS uses a 5-point Likert scale with extremity anchors identical to the UTAUT2 questionnaire.

#### Final Questionnaire

After participants had undergone both interventions and completed the questionnaires, they were asked which intervention should be used to improve clinicians’ acceptability and perceived usability towards the pressure-sensing glove system in future investigations. Potential responses included “video only,” “practice session only,” “both interventions,” “none of the interventions” or “at the clinician’s discretion.” Using open-ended questions, participants could provide comments regarding how the pressure-sensing glove system or the facilitating interventions could be improved.

### Analysis

A descriptive analysis of the participants’ characteristics was first carried out. A thematic content analysis was then performed on responses to the open-ended questions, grouping answers related to the same themes. Likert scale scores were dichotomized using cut-offs defined by the research team to compare proportions of participants who agreed (i.e., checked the options “strongly agree” or the 1 just before) with those who disagreed or were neutral (i.e., checked 1 of the other options) in relation to the acceptability and usability statements. Half of the F-SUS based questionnaire statements are inverted and thus dichotomization was undergone between participants who disagreed (i.e., checked the options “strongly disagree” or the 1 just before) and those who agreed (i.e., checked 1 of the other options including the neutral response). As the Chi-square test revealed no difference in acceptability at baseline between chiropractors and chiropractic students (*p* values > .05), results of both groups were combined for further analyses. McNemar tests were computed to compare the effects of the facilitating interventions on the proportion of participants who agreed and disagreed to each of the usability statements, while Cochran’s Q test was used to compare the proportion between the 3 time points (baseline and following each intervention). When a significant result was obtained from the Cochran’s Q test, Dunn’s post-hoc tests were performed. SPSS software (IBM SPSS Statistics version 27.0.0.0) was used, and significance was set at *P* < .05.

## Results

### Participants

Overall, 16 participants (8 chiropractors and 8 chiropractic interns) took part in this study (56% females) with a median age of 28 years old (range 24-57 years). There was no missing data.

### Facilitating Intervention Effects on General Opinion

The open-ended questions revealed that, at baseline, 7 participants (44%) reported that they believed the system could be relevant for use in private practice with patients to quantify SMT force-time characteristics and potentially improve the treatment provided to their patients. Eight participants (50%) said they did not consider the use of this system relevant for purposes other than research and teaching. One participant (6%) was unsure whether the system should be used outside research. Asked specifically about use for research purposes, 3 participants (19%) reported that they would not hesitate to use the sensing glove system while treating patients for research purposes. Following the video, 1 participant (6%) noted that they would like to practice with the sensing glove system on a manikin or a colleague before using it on a patient, although they had not expressed this need at baseline. One participant (6%) was initially concerned about the risk of injury to the patient or themselves. Following the practice session, the participant indicated that they were now confident in the system’s safety. Another participant (6%) mentioned a decrease in their fear of tactile sensitivity loss following the practice session, while another (6%) noted that the system was less cumbersome and difficult to use than they had initially thought. Other reasons for participants’ reluctance to use the system were perceived difficulty in using the system, system accessibility (i.e., cost), loss of direct touch or accuracy, patient preference not to use the system, concerns about patient comfort, lack of time, perceived decrease in treatment efficacy, and lack of appropriateness on babies.

### Facilitating Intervention Effects on Acceptability

Table 1 presents the number and percentage of participants who agreed to each statement in the acceptability questionnaire. The percentage of participants who agreed that learning to use the sensing glove system was easy for them was significantly higher immediately after the practice compared with the participants’ baseline perception (50% vs. 100%, *P* = .003). No significant effect of the video was observed for this statement (*P > .*05). A significant increase in the percentage of participants who felt that the sensing glove system was easy to use was observed following practice session compared with baseline perception (69% vs.100%, *P = .*04), but not following the video (69% vs.75%, *P* = 1.00). Both the video (75%, *P = .*03) and the practice session (81%, *P = .*01) resulted in a significant increase from the baseline (31%) in the percentage of participants who agreed that they had the necessary knowledge to use the sensing glove system. Finally, all participants agreed that it would be easy for them to become skillful at using the sensing glove system after participating in the practice session compared with 69% at baseline (*P = .*02) and following the video (*P = .*02).

**Table 1:** Percentage of Participants who Agreed to Each Acceptability Statement at Baseline and Following Facilitating Interventions

Survey item	Baseline % (N)	Post video % (N)	Post practice % (N)	Cochran’s Q test with p-value	Dunn’s post-hoc test p values
I find the sensing glove system useful in clinical research.	94% (15)	94% (15)	100% (16)	χ^2^ = 0.00, *P* = .61	–
[I think that] Learning how to use the sensing glove system is easy for me.	50% (8)	81% (13)	100% (16)	χ^2^ = 10.89, *P = .*004	*P = .*003^a^
My [potential] interaction with the sensing glove system is clear and understandable.	69% (11)	75% (12)	100% (16)	χ^2^=5.25, *P = .*07	–
[I think that] I [would] find the sensing glove system easy to use.	69% (11)	75% (12)	100% (16)	χ^2^ = 7.00, p = 0.03*	*P = .*04^a^
It is easy for me to become skillful at using the sensing glove system.	69% (11)	69% (11)	100% (16)	χ^2^ = 10.00, *P = .*01*	*P = .*02^a^*P = .*02^c^
People who I value in the chiropractic profession think that I should use the sensing glove system.	19% (3)	19% (3)	19% (3)	χ^2^ = 0.00, p = 1.00	
I have the necessary knowledge to use the sensing glove system.	31% (5)	75% (12)	81% (13)	χ^2^ = 10.36, *P = .*01*	*P = .*01^a^*P = .*03^b^
I can [will be able to] get help from others when I have difficulties using the sensing glove system.	88% (14)	88% (14)	94% (15)	χ^2^ = 1.00, *P = .*61	
[I think that] Using the sensing glove system is fun.	81% (13)	75% (12)	94% (15)	χ^2^ = 4.67, *P = .*10	
[I think that] Using the sensing glove system is enjoyable.	75% (12)	56% (9)	75% (12)	χ^2^ = 4.50, *P = .*11	
[I think that] Using the sensing glove system is very entertaining.	69% (11)	69% (11)	88% (14)	χ^2^ = 3.60, *P = .*17	
I intend to participate in research projects with the sensing glove system in the future.	75% (12)	63% (10)	75% (12)	χ^2^ = 1.33, *P = .*51	

Statement adaptations for baseline assessment are in square brackets.

⁎ Significant Cochran Q test.

a Significant difference between baseline and practice session.

b Significant difference between baseline and video.

c Significant difference between video and practice session.

At baseline, most participants already agreed that the sensing glove system would be useful in clinical research (94%), that it would be possible to get help with the system (88%) and that the use of the system would be fun (81%) and enjoyable (75%). No changes in these percentages were observed following the video or the practice session (*p values*> .05). Eighty-one percent of the participants did not agree that people they value in the chiropractic profession thought that they should use the sensing glove system and none of the interventions had an effect on this item (*P > .*05).

### Facilitating Intervention Effects on Perceived Usability

Significant differences between interventions in terms of the percentage of participants were only observed for 2 perceived usability statements (Table 2); all participants agreed that the system was easy to use once they had completed the practice session, compared with 63% of participants following the video (*P = .*03). The usability of the system was confirmed by most participants, with the exception of 1 statement. Indeed, most participants (12 following the video-session and 9 following the practice session) felt that using the system requires the support of a technical person.

**Table 2:** Percentage of Participants who Agreed With the Usability Statements Following Both Facilitating Interventions

Survey item	Post video % (N)	Post practice session % (N)	McNemar test with p-value
I think that I would like to use this system frequently.	63% (10)	56% (9)	χ^2^ = 0.00, *P* = 1.00
*I found the system unnecessarily complex.*	6% (1)	0% (0)	χ^2^ = 0.00, *P* = 1.00
I thought the system was easy to use.	63% (10)	100% (16)	χ^2^ = 4.17, *P = .*03*
*I think that I would need the support of a technical person to be able to use this system.*	75% (12)	56% (9)	χ^2^ = 1.33, *P = .*25
I found the various functions in this system were well integrated.	69% (11)	100% (16)	χ^2^ = 3.20, *P = .*06
*I thought there was too much inconsistency in this system.*	19% (3)	12% (2)	χ^2^ = 0.00, p = 1.00
I would imagine that most people would learn to use this system very quickly.	69% (11)	88% (14)	χ^2^ = 0.80, *P = .*38
*I found the system very cumbersome to use.*	19% (3)	0% (0)	χ^2^ = 1.33, *P = .*25
I felt very confident using the system.	44% (7)	88% (14)	χ^2^ = 5.14, *P = .*02*
*I needed to learn a lot of things before I could get going with this system.*	19% (3)	0% (0)	χ^2^ = 1.33, *P = .*25

⁎ Significant difference between following the video and following the practice session. Inverted statements are in italic.

### Participants’ Opinions on the Interventions

Overall, 14 participants (88%) considered that a combination of both interventions should be used to increase clinicians’ acceptability and usability of the use of the sensing glove system with patients. The other 2 participants (13%) suggested that a practice session would be enough. Five participants (31%) mentioned that the study and/or the sensing glove system were very interesting. One participant (6%) highlighted that seeing their force-time curve during the practice session allowed them to identify an aspect of their SMT force-time curve to improve. Other comments were that such a system will greatly help students and clinicians improve their techniques, that they looked forward to future developments, and that they were enthusiastic about using the system in their practice to measure their manual therapies force for each patient based on their condition. Two participants (13%) identified the loss of tactile sensation due to the sensor and the glove as a limitation of the sensing glove system is. Another participant (6%) noted that the sensor fabric could be uncomfortable for the patient.

## Discussion

This study had 2 primary objectives; the first 1 was to assess the acceptability of a sensing glove system designed to measure the force-time characteristics of SMT administered by chiropractors and chiropractic students, the second 1 was to compare the effectiveness of 2 interventions aimed at enhancing participants’ acceptability and perceived usability of the system. The results indicate that, in the absence of any facilitating interventions, the acceptability of the sensing glove system was primarily influenced by concerns about impact on treatment execution, difficulty of using the system, and perceived relevance of its use. Among the acceptability-enhancing interventions, a 20-minute hands-on practice session proved more effective in increasing the system’s acceptability and perceived usability than a brief video demonstration. These results have significant implications for the future development and implementation of force-sensing technology in chiropractic care.

This study represents the first investigation of end-user acceptability and perceived usability of the use of a force-sensing system in patient care. As a result, direct comparisons to previous investigations are limited. A recent scoping review identified various factors that may influence the use of force-sensing systems in manual therapy research, education, and clinical practice.^6^ These factors include the system’s metrological properties, cost, durability, user-friendliness, versatility, clinician and patient comfort, disturbance of the device during technique execution, and the system’s ability to provide feedback.^6^ Some of these factors were also reported as concerns by the participants of the present study. For instance, participants mentioned concerns about losing tactile sensation due to the sensor and glove, as well as potential discomfort for the person on whom the system is used due to the sensor fabric. Following the practice session, these apprehensions decreased, supporting the idea that even a brief experience with the sensing glove system can improve its acceptance among manual therapists. In the short term, however, thoughts need to be given to the fabric to which the sensing elements are attached, in order to minimize any potential interference between the clinician’s hand and the patient’s back.

Based on the UTAUT2 model proposed by Venkatesh,^22^ the user’s level of experience with the technology investigated, or with similar technology, would influence acceptability by moderating their behavioral intention. Previous studies have demonstrated that users’ experience with a new technology can significantly impact their perceptions of its acceptability and ease of use. This phenomenon has been observed in various fields, including mobile applications^26^ and the use of social robots with vulnerable populations.^27^ As far as usability is concerned, this measure is highly context-specific, and as such, experience with other similar technologies have limited influence.^25^ In the present study, it should be noted that most participants initially expressed positive opinions towards the sensing glove system, considering it useful and enjoyable. However, the integration of a practice session significantly improved the acceptability and perceived usability of the system. This was evidenced by the participants’ improved perception of the system’s ease of use, ease of learning, and their confidence in operating it effectively. Open-ended questions provided valuable insights, indicating that the practice session reassured participants regarding the system’s safety, manageability, and their ability to use it effectively, while also addressing concerns about potential loss of tactile sensitivity. Although viewing a video demonstration also had a positive impact on the system’s acceptability, its significant effect was observed mainly in the participants’ knowledge of the system’s functionality and ease of use. The evaluation of a short video demonstration aimed to increase participants’ knowledge about the system, an important factor in the implementation of new practices among healthcare professionals.^28^ However, based on the current study, it appears that this intervention had limited additional benefit beyond reading a standard information sheet that participants received at baseline when deciding whether to consent to participate in the study. As demonstrated by these results, allowing chiropractors or chiropractic students to engage in a structured practice session with the force-sensing technology and giving them the opportunity to ask questions could significantly reduce hesitation and increase their willingness to use the system with their patients.

The integration of force-sensing technology into chiropractic education is still relatively recent.^29^^,^^30^ Consequently, the majority of chiropractors who participated in the current study (5 out of 8) had not been exposed to such technology during their training or practice. In contrast, most of the chiropractic students in this study (7 out of 8) had had prior experience using a force-feedback tool as a part of their curriculum. This suggests that the next generation of chiropractors may be more inclined to adopt this type of technology to enhance patient care. However, the hypothesis was not fully confirmed, as the proportions of chiropractic students agreeing and disagreeing with the acceptability statements did not significantly differ from those of the chiropractors. To facilitate the adoption of the force-sensing system into future clinical practice among students, its implementation in the University outpatient clinic could be a valuable approach, but further research is needed to confirm this.

### Research Perspectives

Before implementing a force-sensing technology into clinical practice, further research is needed to explore how it can improve patient care efficacy and safety. In these future investigations, it will be crucial to consider offering participants the opportunity to try out the system and engage in discussions with the research team. These strategies, in addition to the research information sheet, could strengthen clinicians’ willingness to participate by easing their reluctance to use the device with their patients and improving their understanding of how the data recorded by the device could be valuable for their practice. Furthermore, despite the accessibility of a practice session, providing a video could be a cost-effective option.

### Strengths

The main strength of this study is the use of standardized questionnaires to evaluate participants’ opinion on the sensing glove system acceptability and usability. Inclusion of open-ended questions also provided participants the opportunity to explain their beliefs about the factors that would limit their use of the system during patient care.

### Limitations

First, the order of the interventions was randomized between participants, but it cannot be excluded that the first intervention proposed to participants might have influenced their evaluation following the second intervention. Moreover, the psychometric properties of the Canadian French version of the UTAUT2 questionnaire have not been evaluated, and the impact of selecting only certain items or modifying items for baseline acceptability assessment (i.e., before any facilitating interventions) is unknown. Although both questionnaires are validated and widely used to assess the acceptability and perceived usability of new rehabilitation-related technology, to our knowledge, their sensitivity to change has not been assessed. Finally, since the sample size was determined based on the exploratory nature of this study, it may be underpowered, which means that the additional benefits of including practice sessions over a simple video need to be confirmed in a larger population of chiropractors and chiropractic interns.

### Future Studies

Future studies should consider offering potential participants the opportunity to try out the system and discuss with the research team, while providing research information, to increase their participation rate and confidence in using the device with their patients. Despite the possibility of a practice session, providing a video could be a cost-effective option. Overall, these results have the potential to foster the adoption of force-sensing technology by chiropractors, ultimately improving the quality of care for their patients.

## Conclusion

This study provides valuable insights on the impact of 2 acceptability-facilitating interventions on the acceptability and perceived usability of a sensing glove system by chiropractors and chiropractic students. To successfully integrate this technology into clinical practice and enhance patient care efficacy and safety, further investigations are necessary.

## Contributorship Information

*Concept development* (provided idea for the research)**:** MAM, IP

*Design* (planned the methods to generate the results)**:** IP, MD, AKB

*Supervision* (oversight, organization and implementation)**:** IP

*Data collection/processing* (experiments, organization, or reporting data)**:** MAM, IP

*Analysis/interpretation* (analysis, evaluation, presentation of results)**:** MAM, IP, AKB, MD

*Literature search* (performed the literature search)**:** MAM, IP

*Writing* (responsible for writing a substantive part of the manuscript)**:** MAM, IP, AKB

*Critical review* (revised manuscript for intellectual content)**:** MD

*Other* (list other specific novel contributions):

## Appendix Supplementary materials

Image, application 1
