# Supplementary material for: Effects of 2 Interventions on the Acceptability and Usability of a Sensing Glove for Measuring Force-Time Characteristics of Chiropractic Spinal Manipulative Therapy: A Crossover Study
Source: J Chiropr Med. 2025 Sep 10;24(1-4):44–53. doi: 10.1016/j.jcm.2025.08.002 (PMC12804030; doi:10.1016/j.jcm.2025.08.002)
Supplement: Supplementary file 1 [file mmc1.pdf]

Vidéo explicative

Dans le cadre du projet :
Comparer les effets immédiats de deux stratégies sur l'acceptabilité et la perception d'utilisabilité d'un système de gants instrumentés en recherche clinique par des internes en chiropratique et des chiropraticiens

Chercheuse principale
Marie-Andrée Mercier
Étudiante au doctorat en chiropratique à l'UQTR

Sous la supervision de
Isabelle Pagé DC PhD
Andréanne K. Blanchette PT, PhD

En collaboration avec Martin Descarreaux DC, PhD

GRAN Groupe de Recherche sur les Affections Neuromusculosquelettiques

Cirris Centre interdisciplinaire de recherche en réadaptation et intégration sociale

Fondation Chiropratique du Québec

Université Laval

UQTR Université du Québec à Trois-Rivières

CER-21-278-07.26

Objectifs de la présentation

Cette présentation de 7 minutes vise à vous faire découvrir le nouveau système de gants instrumentés disponible à l'UQTR, et qui sera utilisé lors de futurs projets de recherche.

Vous en apprendrez davantage sur la biomécanique de la manipulation et de la mobilisation vertébrale, le mode de fonctionnement du système de gants instrumentés ainsi que sur sa pertinence en recherche clinique.

À la fin de cette présentation, une vidéo contenant une démonstration de l'utilisation du système de gants instrumentés vous sera présentée.

UQTR

Pourquoi est-il nécessaire de mieux connaître la biomécanique des thérapies manuelles ?

UQTR

Pourquoi ?

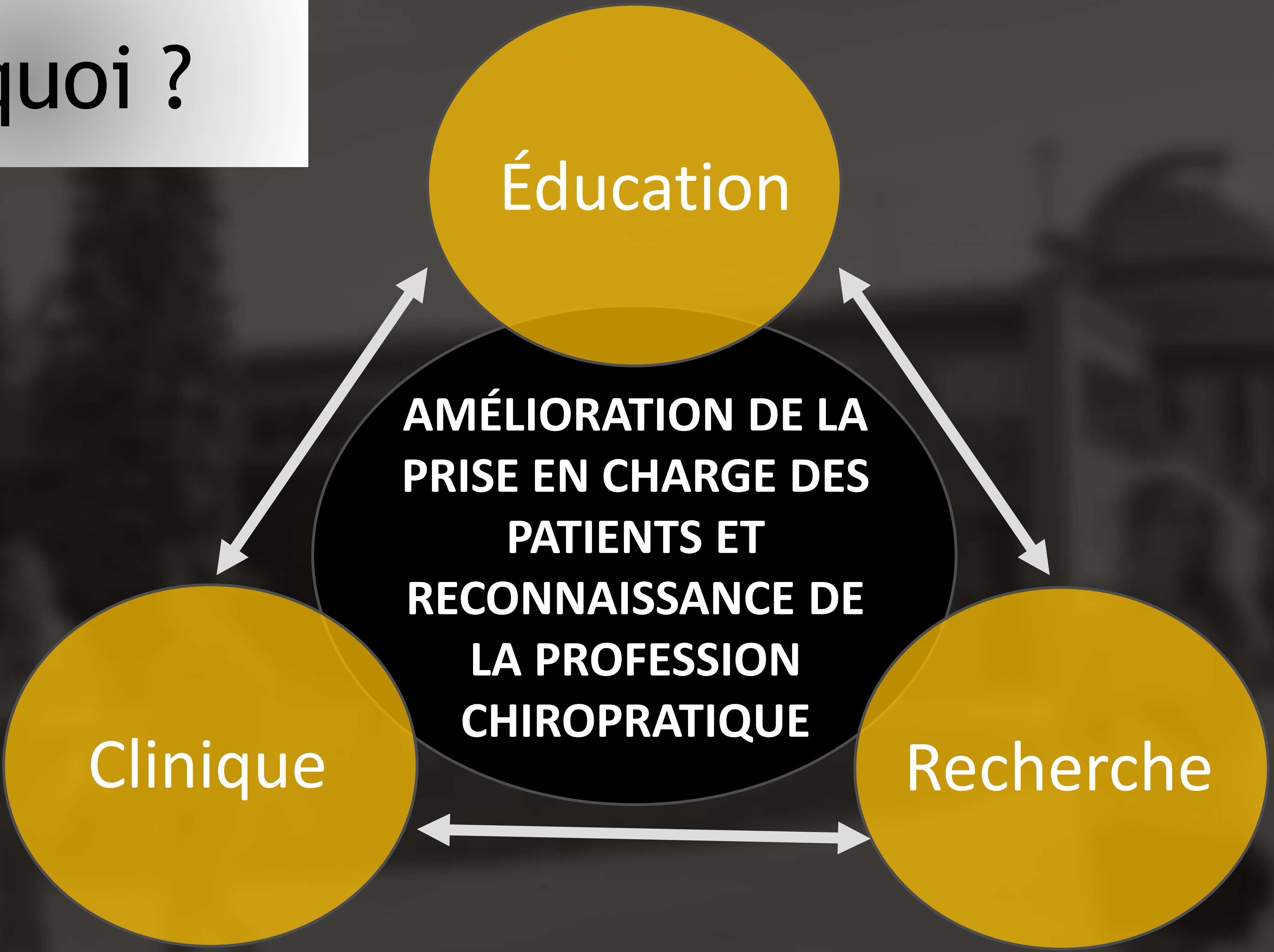

UQTR

Pourquoi ?

Recherche

- Déterminer les mécanismes qui sous-tendent les effets cliniques des manipulations et mobilisations vertébrales.
- Investiguer la sécurité des manipulations et mobilisations vertébrales.
- Comprendre les adaptations des techniques utilisées par les chiropraticiens en fonction des patients.

Éducation

- Intégration d’objectifs quantitatifs (par ex., force ou vitesse à atteindre) lors des cours de techniques chiropratiques.
- Développement d’outils de rétroaction pour les étudiants.

Clinique

- Développement d’outils pouvant être utilisés lors de la prise en charge de certains patients: CNESST, SAAQ, cas chroniques.
- Adaptation du consentement éclairé aux soins.

Comment mesure-t-on les paramètres biomécaniques ?

Paramètres biomécaniques

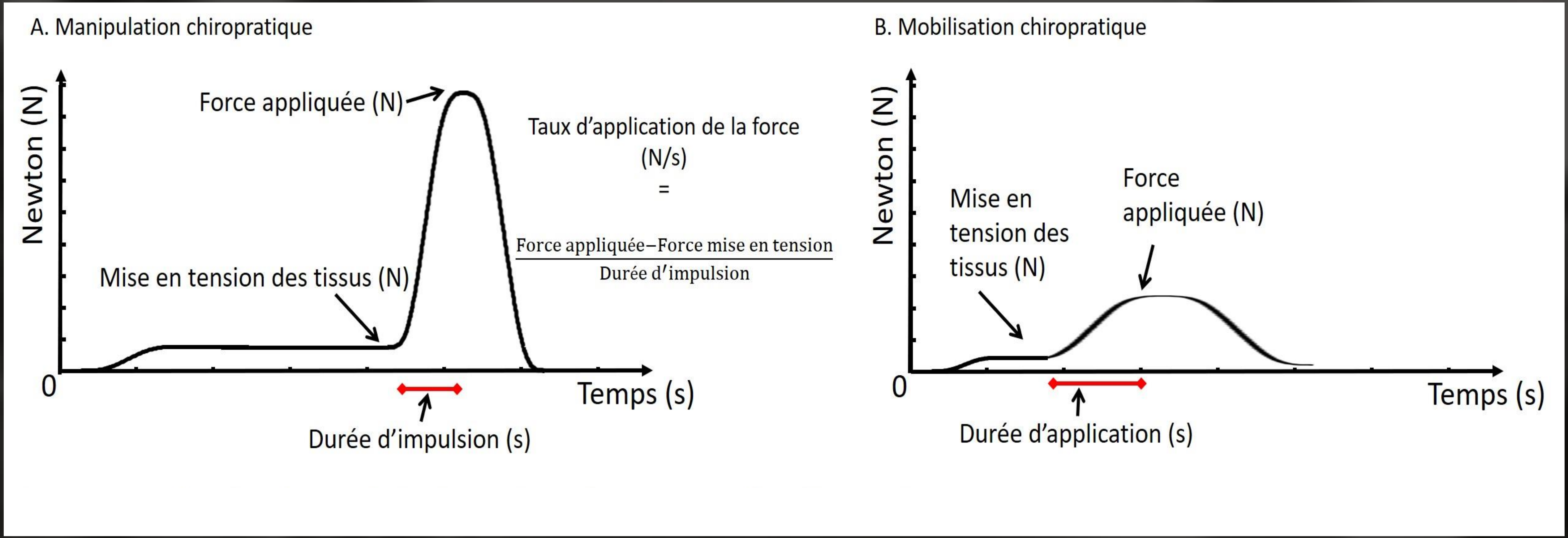

Projets réalisés

Au cours de la dernière décennie, de nombreux projets en lien avec la biomécanique de la manipulation ou de la mobilisation vertébrale ont été effectués :

- Influence des paramètres biomécaniques dans :
  - La stimulation des réflexes musculaires paravertébraux et le déplacement vertébral
  - Les effets cliniques chez les patients ayant des douleurs thoraciques
  - La modulation de la transmission des signaux nociceptifs
  - La transmission de la force à travers le thorax
  - Les contraintes induites aux tissus vertébraux

- Évaluation de l’apprentissage des manipulations vertébrales des étudiants en chiropratique.

UQTR

Appareils disponibles à l'UQTR

Boîtier instrumenté

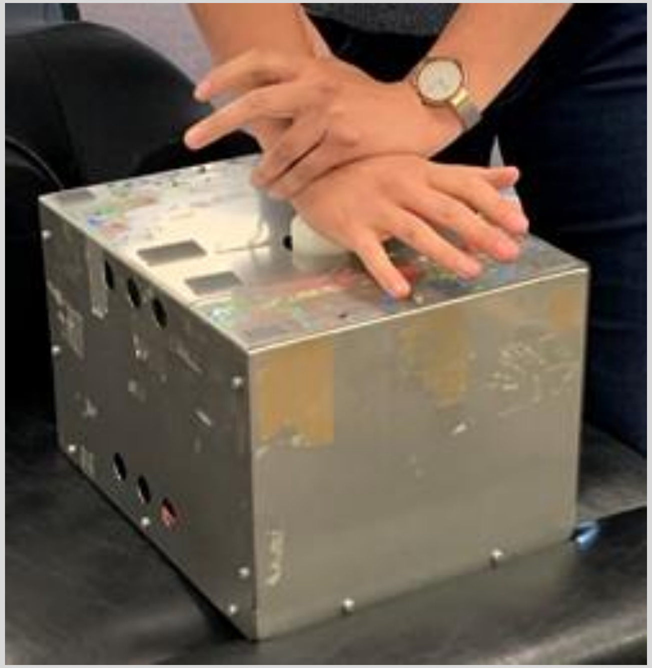

Appareil robotisé

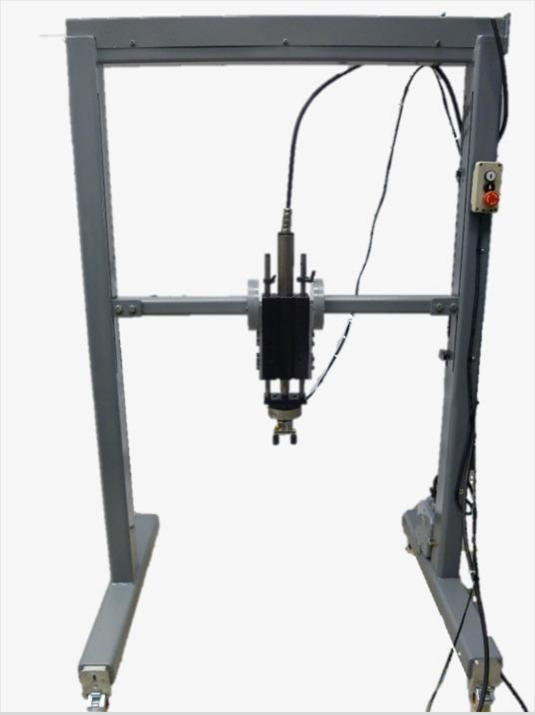

Table instrumentée

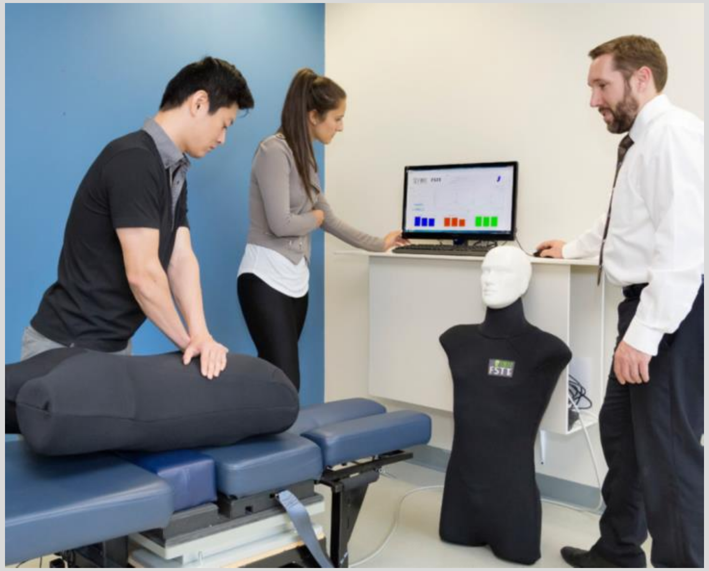

Qu'est-ce que le système de gants instrumentés ?

Système de gants instrumentés

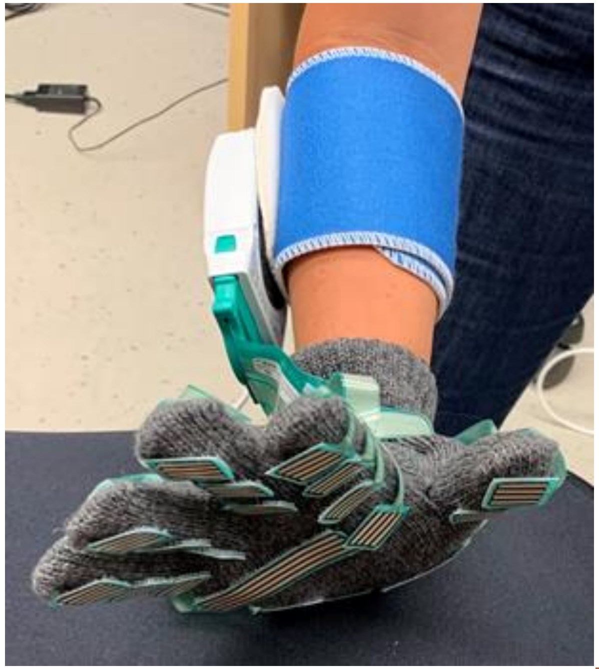

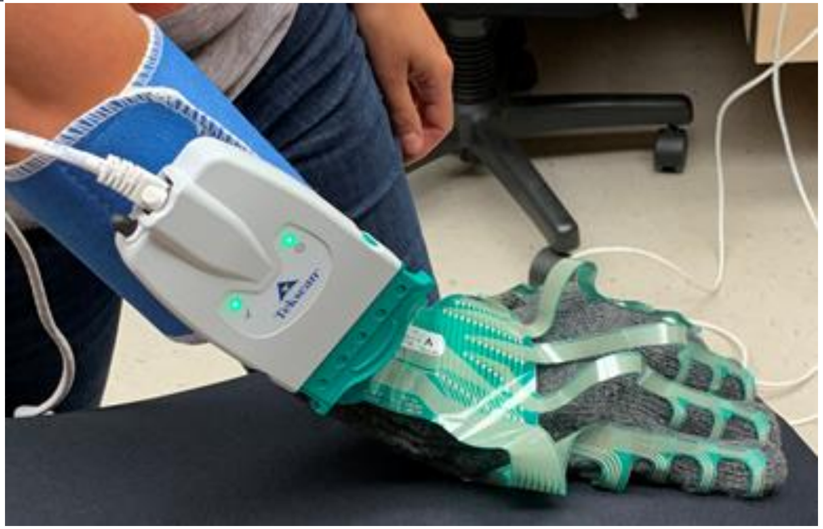

Tekscan™

- Système disponible à l'UQTR depuis le printemps 2021
- Collaboration avec un chercheur du département de génie électrique et génie informatique pour tirer le maximum du système !
- Chaque gant comporte 349 points mesurant la pression/force en fonction du temps.
- Mesure en contexte clinique:
  - Transportable
  - Facile d'utilisation
  - Mesure à la main du clinicien
  - Possibilité de rétroaction instantanée !

Démonstration du fonctionnement des gants

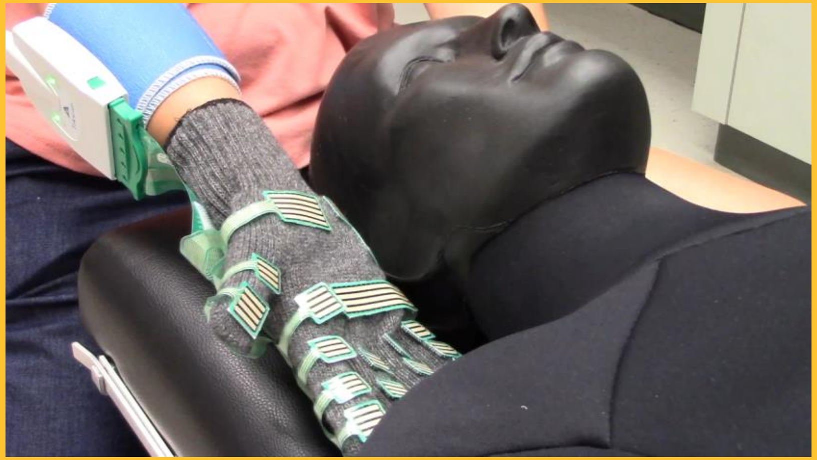

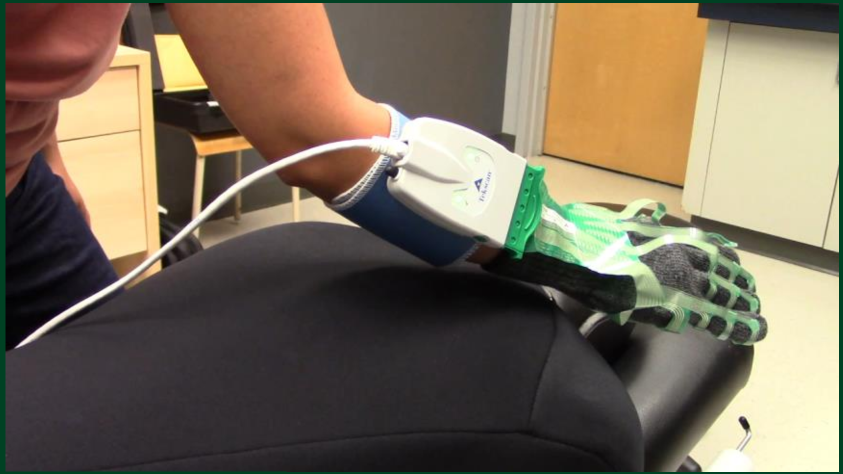

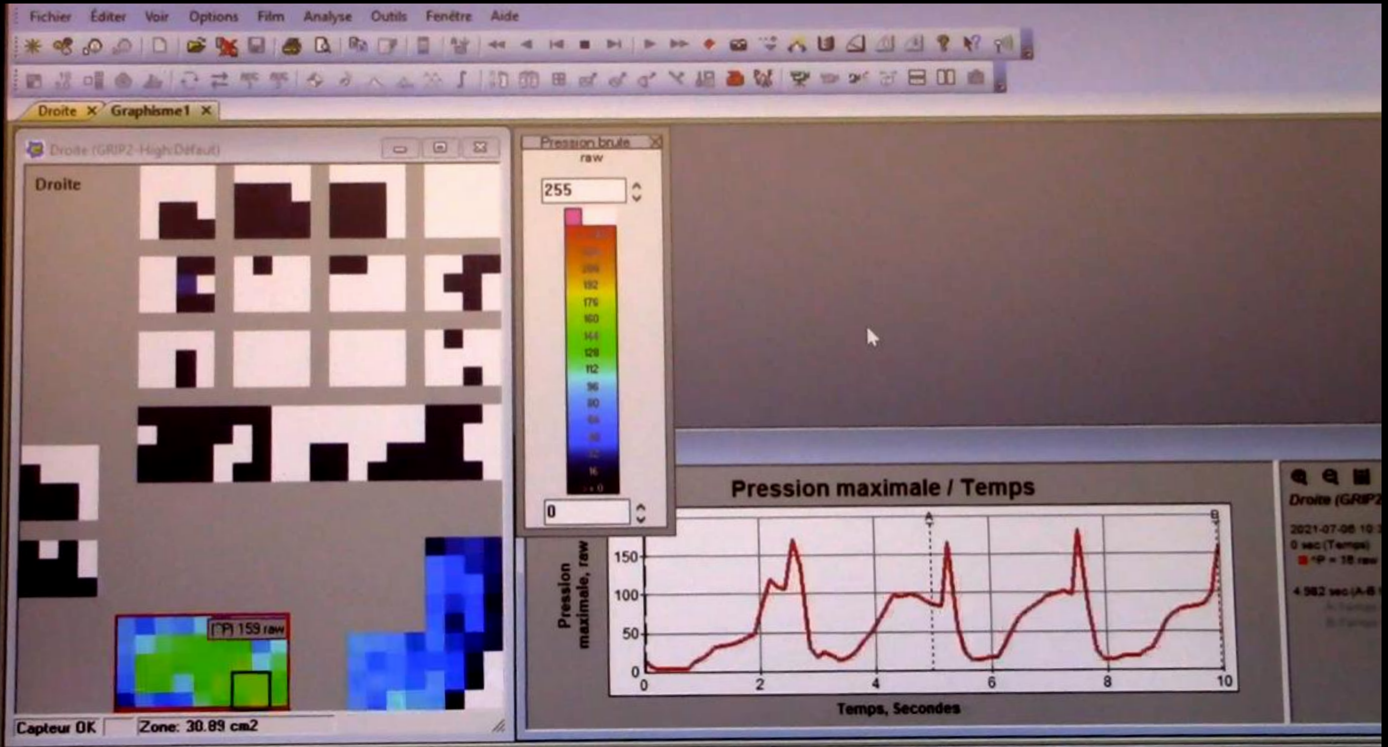

UQTR

Merci

819-376-5011
#3885

Isabelle.page1@uqtr.ca
